# Combinatorial therapeutic strategies for enhanced delivery of therapeutics to brain cancer cells through nanocarriers: current trends and future perspectives

**DOI:** 10.1080/10717544.2022.2069881

**Published:** 2022-05-09

**Authors:** Xiande Wang, Cheng Wu, Shiming Liu, Deqing Peng

**Affiliations:** a Department of Neurosurgery, Hangzhou Medical College Affiliated Lin’an People’s Hospital, The First People’s Hospital of Hangzhou Lin’an District, Hangzhou, China; b Cancer Center, Department of Neurosurgery, Zhejiang Provincial People’s Hospital (Affiliated People’s Hospital, Hangzhou Medical College), Hangzhou, China

**Keywords:** Brain cancer, nanocarriers, blood-brain barrier, blood-cerebrospinal fluid barrier, dual-targeting

## Abstract

Brain cancer is the most aggressive one among various cancers. It has a drastic impact on people's lives because of the failure in treatment efficacy of the currently employed strategies. Various strategies used to relieve pain in brain cancer patients and to prolong survival time include radiotherapy, chemotherapy, and surgery. Nevertheless, several inevitable limitations are accompanied by such treatments due to unsatisfactory curative effects. Generally, the treatment of cancers is very challenging due to many reasons including drugs’ intrinsic factors and physiological barriers. Blood-brain barrier (BBB) and blood-cerebrospinal fluid barrier (BCSFB) are the two additional hurdles in the way of therapeutic agents to brain tumors delivery. Combinatorial and targeted therapies specifically in cancer show a very promising role where nanocarriers’ based formulations are designed primarily to achieve tumor-specific drug release. A dual-targeting strategy is a versatile way of chemotherapeutics delivery to brain tumors that gets the aid of combined ligands and mediators that cross the BBB and reaches the target site efficiently. In contrast to single targeting where one receptor or mediator is targeted, the dual-targeting strategy is expected to produce a multiple-fold increase in therapeutic efficacy for cancer therapy, especially in brain tumors. In a nutshell, a dual-targeting strategy for brain tumors enhances the delivery efficiency of chemotherapeutic agents via penetration across the blood-brain barrier and enhances the targeting of tumor cells. This review article highlights the ongoing status of the brain tumor therapy enhanced by nanoparticle based delivery with the aid of dual-targeting strategies. The future perspectives in this regard have also been highlighted.

## 1. Introduction

Brain cancer is aggressive and devastating one among various cancers. It has a severe impact on people's lives due to failure in treatment efficacy of this cancer (Zottel et al., 2019). Brain cancer is characterized by life-threatening and worsened symptoms that have effects on quality of life (Butler et al., 2019). The pathophysiology of brain cancer is governed by genes mostly including, tumor suppressor genes, DNA repair genes, and proto-oncogenes (Bian et al., 2018; Xu et al., 2020). Various strategies used to relieve pain in brain cancer patients and prolong life survival time include radiotherapy, chemotherapy, and surgery (Coomans et al., 2019; Kahalley et al., 2019). Nevertheless, several inevitable limitations are produced due to unsatisfactory curative effects. In the context of surgery, the physical eradication of brain tumors is quite challenging due to the distinguishing and separation of tumors from normal brain tissues (Zeineldin et al., 2020). In the case of radiotherapy, a low therapeutic efficacy outcome is faced because inside the hypoxic tumor microenvironment the brain tumor cells lack the desired sensitivity to ionizing radiations (Aldape et al., 2019). Similarly, in chemotherapy, many factors pose hurdles in the efficient delivery of drugs such as nonspecific targeting, insufficient physiological stability, and blood-brain barrier impedance (Ganipineni et al., 2018; Yu et al., 2019).

**Figure 1.**
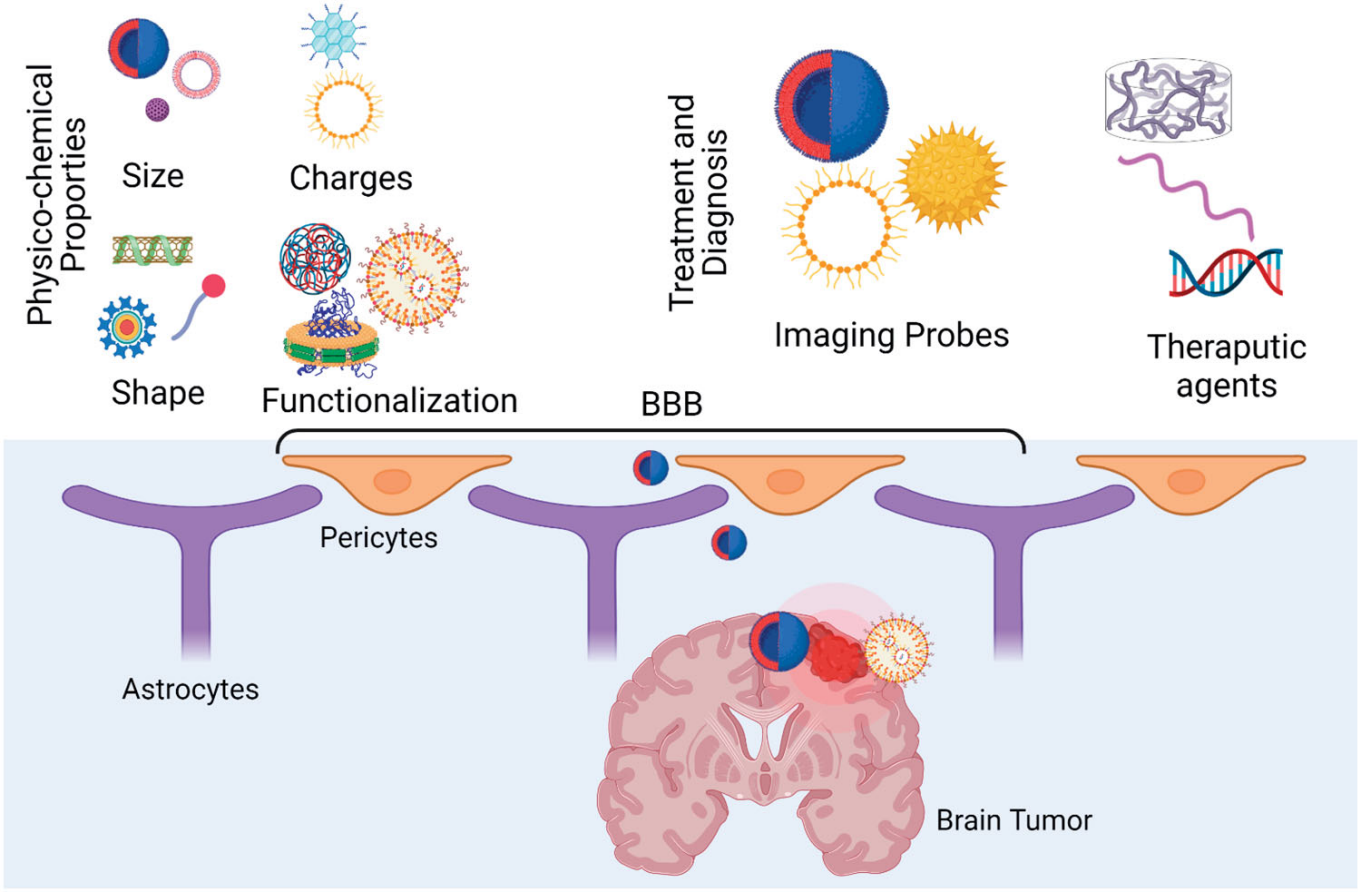
Factors affecting the transport of substances between BBB and tumor cells.

The blood-brain barrier (BBB) is a complex, compact, and specific dynamic interface that exists between the central nervous system (brain and spinal cord) and blood capillaries (Gupta et al., 2019). BBB is mainly composed of cerebral capillary endothelial cells that are densely packed and encompassed by astrocytes and endothelial cells through basilemma (Coelho‐Santos & Shih, 2020). In addition, BBB shows properties similar to semi-permeable membranes due to the presence of proteins in the interstitial fluids (Oddo et al., 2019). Thus, such chemistry of BBB restricts the free exchange of materials between brain tissue and blood, protecting brain tissues from the harmful effect of foreign substances. However, despite this protecting effect, BBB also avoids the penetration of therapeutic agents into the brain tissue making the treatment of brain disorders a challenging scenario. Recent strategies employed to cope with this issue include osmotic destruction (Li et al., 2021), intrathecal injection, nasal administration (Bellettato & Scarpa, 2018), and ultrasound interferences of drugs (Idbaih et al., 2019). Nevertheless, such approaches result in BBB damage, trauma, and biotoxicity (He et al., 2018). Thus to ensure safe and effective therapeutic delivery to brain tumor cells, more convenient and safe strategies are needed. A schematic illustration of BBB and penetration of agents across, and factors affecting the agents’ penetration is depicted in Figure 1.

The delivery of therapeutic agents from nano – the platform is among the explored strategies that have targeting the potential for brain tumors. Conventional delivery systems for cancer therapeutics are facing the problems of treatment failures and side effects due to the off-target release of the drugs. In addition, drug resistance of tumor cells and the BBB impedance aid in the hurdles for successful brain cancer treatment (Ni et al., 2019). The application of targeting strategies particularly from nano scaffolds has opened new horizons in the domain of oncology (Tang et al., 2019). Due to nano size, the nanocarriers easily penetrate the BBB and reach the target site. For targeting brain tumors, the explored nanomaterials include but are not limited to, liposomes, dendrimers, micelles, gold nanoparticles, and polymeric nanoparticles (Teleanu et al., 2018). These delivery systems ensure safe drug dosing, control drug loading/release, provide a noninvasive approach, give stability, and prolong shelf life (Teleanu et al., 2018). Overall, nano particles-based delivery of chemotherapeutic agents to brain tumors significantly penetrates the BBB, however, the accumulation of chemotherapeutic agents at the target site is a separate scenario.

Blood-brain barrier and blood-cerebrospinal fluid barrier are the main hurdles in the way of therapeutic agents are delivery to brain tumors (Barbara et al., 2017). Therefore, the delivery of therapeutic molecules to the brain usually requires energy-dependent-transport mechanisms to transport nanomaterials and drugs across the BBB. Various strategies for improving CNS delivery including local injections or surgical openings of BBB have been developed to facilitate targeted delivery and improve drugs’ permeability to the brain (Pardridge, 2007). Intra-cerebroventricular and intracerebral administration, intra-nasal delivery, and blood-to-brain delivery through transient disruption of BBB mediated via chemical, biological, or physical stimuli e.g., mannitol, zonula occludens toxin, ultrasound, and magnetic heating have also been reported strategies, however, these approaches are being dangerous, unsuitable for most brain drugs and diseases as well as pose high cost (Li et al., 2017). Regulated trans-cytosis for enhancing the permeability of therapeutics or other substances seems an appealing approach for enabling the transport of macromolecular entities which are not usually possible through normal routes (Mäger et al., 2017). A dual-targeting strategy is a versatile way of chemotherapeutics delivery to brain tumors that gets the aid of combined ligands and mediators that cross the BBB and reaches the target site efficiently (Peng et al., 2018). In a nutshell, a dual-targeting strategy for brain tumors enhances the delivery efficiency of chemotherapeutic agents via penetration across blood-brain barrier and enhances the targeting of tumor cells. This review article highlights the ongoing status of the brain tumor therapy enhanced by nanoparticles based delivery with the aid of combinatorial targeting strategies, especially dual-targeting. The future perspectives in this regard have also been highlighted.

## 2. Brain cancer and blood-brain barrier

**Figure 2.**
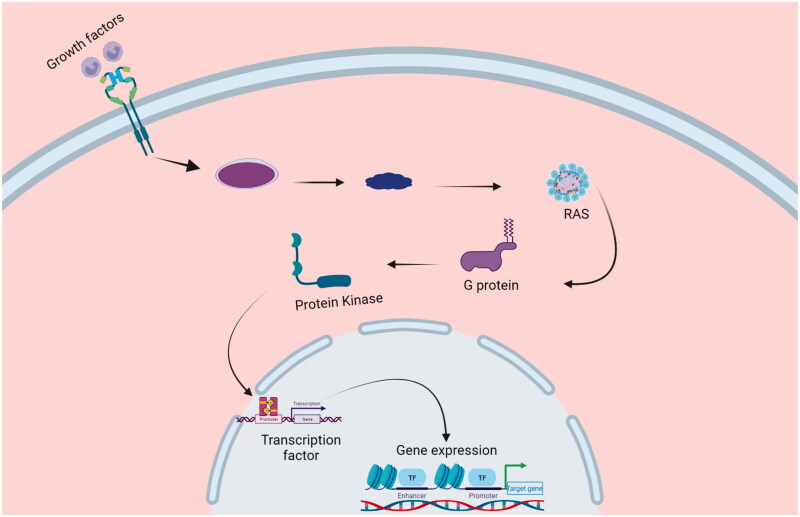
Signal transduction cascades are involved in cell cycle proliferation.

Brain cancer is one of the fatal cancers that develop in the brain or spinal cord (Alemany et al., 2021). Brain cancer is characterized by tumors with main symptoms of difficulty in speech, coordination problems, memory loss, mood swings, and seizures (Raghavapudi et al., 2021). Depending upon growth rate, nature and progression stage, the brain tumors are categorized into various types but they may be benign or malignant in nature (Carey-Ewend et al., 2021). Benign brain tumors are characterized by their slow progression with distinct borders and invade neighbor healthy cells infrequently. Examples are pituitary tumors and meningiomas. While malignant brain tumors such as astrocytoma and oligodendroglioma exhibit rapid progression rate, fuzzy borders, and readily attack the neighboring healthy cells (Butler et al., 2019; Tandel et al., 2019). From a progression rate point of view, brain cancer ranges from stage 0 to 4 (Amin et al., 2020). The pathophysiology of brain cancer is mainly controlled by genes. Mutations in the DNA lead to gene control modifications that result in abnormal brain cell division and ultimately brain cancer (Lu et al., 2019). Brain tumor suppressor genes govern the control of apoptosis (Cell death). Such apoptosis is either induced by the cell itself for suicide cascades or through the reception of death signals from the nearby cells. Thus cell death process could be slowed down by mutation in any of the mentioned pathways (Huang et al., 2018). Also, malfunctioning of the DNA repair genes contributes to brain cancer (Ratnaparkhe et al., 2018). In addition, proto-oncogenes are another group responsible for disturbing the tumor suppressor gene functions and lead indirectly to brain tumors (Batra et al., 2021). In healthy cells, signal transduction cascades are involved in normal brain cell division as shown in Figure 2, which operates the normal cell cycle and any mishaps in normal brain cell cycle leads to the respective cancer.

**Figure 3.**
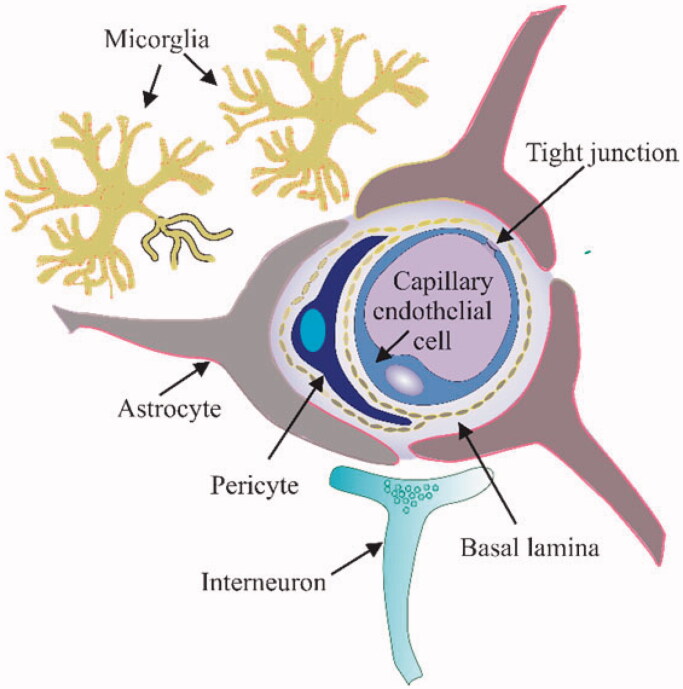
Structure of Blood-Brain Barrier (BBB).

The blood-brain barrier (BBB) is a compact and complex structure due to endothelial cell tight junctions (Reddy et al., 2021). It is comprised of perivascular mast cells, endothelial cells, astroglia, and pericytes as shown in Figure 3 (Sonar & Lal, 2018). BBB lies around the capillaries of the brain and spinal cord thus offering a significant barrier to the circulating systemic drugs and pathogens (Harilal et al., 2020). It produces a high trans-endothelial electrical resistance (TEER i.e., 1500–2000 Ω·cm^2^ compared with 20 Ω·cm^2^ of vascular tissues) which restricts the transport and diffusion of therapeutic drugs to the brain from the blood (Butt et al., 1990). In addition, there are various efflux transporters present on the BBB including p-glycoproteins (P-gps), breast cancer cell resistance proteins and multiple drug resistance proteins which pump out the drugs and restrict their entry to the brain further attenuating drug concentrations in the brain (Qosa et al., 2015). Thus, nearly all free peptides, genes and proteins and around 98% of small molecules cannot penetrate the BBB (Pardridge, 2007). It allows low molecular weight neutral chemotherapeutic drugs while hydrophobic drugs with a molecular weight of less than 500 Dalton (Haumann et al., 2020). In contrast, most chemotherapeutic drugs are charged, hydrophilic and of large molecular weight. For such drugs, BBB is a big hurdle to achieving a constant brain drug concentration (Cao et al., 2020). In starting doses it is quite difficult to maintain the therapeutic effective dose at the tumor site because if BBB is penetrated by such drugs then again it faces the issue of back diffusion (Furtado et al., 2018). An effective therapy strategy for brain tumor needs an effective delivery system to penetrate the BBB and also accumulate in the tumor cells at such concentration that is sufficient for therapeutic outcome. The current research focus is mostly based on BBB as the main hurdle in attaining adequate drug concentration in CNS tumors. Yet, similar other obstacles including blood-CSF and blood-tumor barrier (BTB) exists that impede drug accumulation in brain tumors. Several other factors e.g., increased interstitial pressure, altered intra-tumoral vasculature and peri-tumoral edema also hamper the delivery of drugs to brain tumors (Warren, 2018).

Most of the small lipophilic compounds cross BBB via simple diffusion. Additional processes for the transport of drugs and other substances across BBB include carrier-mediated (facilitated) diffusion, diffusion through aqueous channels, paracellular transport, and active transport (Warren, 2018). Physicochemical characteristics of the drug i.e., hydrogen bonding, lipid solubility, molecular weight, as well as metabolism, cerebral blood flow, degradation, drug clearance from the blood, and protein bindings are the factors that determine the BBB traversing of the drug. The only free unbound drug can cross the BBB through transendothelial diffusion (Banks, 2016). Highly lipophilic, small-sized, low molecular weight and low hydrogen-bonding characteristics of the drug favor its crossing through BBB (Warren, 2018).

## 3. Targeting strategies for cancer treatment

Generally, the treatment of cancer is very challenging due to many reasons. The microenvironment of tumors owe several special characteristics including low extracellular pH, higher interstitial fluid pressure, and irregular vasculature which combined with other cellular events (e.g., efflux transporters’ over-expression, altered molecular targets, and defective apoptotic machineries) pose multidrug resistance (MDR) toward anticancer drugs (Patel et al., 2013). In addition, the majority of the anticancer medications lack the desired pharmacokinetic and physicochemical properties due to less stability, low water solubility, high nonspecific nature, and high toxicities thus becoming inefficient in patients. Therefore, targeted therapies specifically in cancer show a very promising role where nanocarriers’ based formulations are designed primarily to achieve this task (Torchilin, 2007) and several products have been approved. The tumor cells are usually targeted via active or passive targeting strategies. In passive targeting methods, the physicochemical characteristics of nanocarriers and the physiological factors of tumors are employed collectively known as the enhanced permeability and retention (EPR) effect (Maeda et al., 2009; Torchilin, 2011; Pattni & Torchilin, 2015). There is an extensive proliferation in tumor cells requiring large amounts of nutrients thus resulting in expedited blood vessels formation with leaky and irregular vasculature. The spaces in endothelial layers of the capillaries allow nanocarriers and drugs of <500 nm size to extrude into tumors. In addition, there is no proper lymphatic drainage system in tumors that help in the accumulation of drug-loaded nanocarriers in tumor tissues. In certain cases, the nanocarriers are modified with ligands e.g., polyethylene glycol (PEG) to enhance the circulation times of the nanocarriers by avoiding the reticuloendothelial system (RES) and enhance their effects by allowing them more time to accumulate in tumors. Active targeting is accomplished by incorporating alterations in the nanocarriers that not only aid in targeting the tumors but also can overcome the tumor resistance factors (Pattni & Torchilin, 2015). For example, the MDR is generated by the ATP-binding cassette transporters e.g., P-glycoproteins (P-gps) which are mainly responsible for the efflux of anticancer drugs from tumor cells and are present on cell membranes (Patel et al., 2013). To circumvent such drugs efflux transporters, the anticancer drugs are administered in nanocarriers which have the potential to block these P-gps thus improving their anticancer efficiencies (Patil et al., 2010; Patel et al., 2011).

Active targeting of nanocarriers can also be accomplished by conjugating the carriers with targeting ligands such as folate, transferrin, antibodies, and many other ligands (Bae et al., 2012; Sawant et al., 2014; Pattni & Torchilin, 2015). These ligands containing nanocarriers are able to reach their receptors which are over-expressed in certain cancer cells or tissues thus selectively reaching those sites and releasing the payload in target areas. A more recent interesting strategy for tumor targeting is the application of dual-targeted systems which facilitate step-by-step entry of the drugs and nanocarriers into the tumor cells (Sawant & Torchilin, 2011; Koren et al., 2012; Zhu & Torchilin, 2013). It is the delivery of anticancer drugs in nanocarriers’ with their own intrinsic therapeutic potential thus synergistically improving the therapeutic potential of treatment. In this targeted therapeutic strategy, specific cancer proteins and/or biological transduction pathways are blocked thus inducing cancer cells' death by virtue of apoptosis or immune stimulation. Monoclonal antibodies or small molecules inhibitors are used to accomplish such targeted therapies by directly altering specific cell signaling (Pérez-Herrero & Fernández-Medarde, 2015). The combined targeting strategy is the targeted delivery of drug-loaded carriers with molecular specificity providing a directed approach to the target site. The double targeting strategy is the combined spatial-temporal methodologies for spatial placement of the carriers and drugs to specific target sites and then releasing the drug temporally a controlled manner (Kumar et al., 2017; Tewabe et al., 2021).

Dual-targeting strategies can be divided into two main types including (i) that work directly on target structures i.e., soluble factors or cell surface receptors and (ii) that use dual-targeting for delivery of a therapeutically active drug, e.g., effector cells and effector molecules (Kontermann, 2012). Direct actions include neutralization and binding of two receptors or two ligands, neutralization of a ligand and a receptor, activation of two receptors, activation of one receptor and neutralization of another receptor or a soluble factor. It may also include neutralization by binding to different epitopes of one receptor or ligand. Indirect actions include antibody-dependent cell-mediated cytotoxicity (ADCC) and cell dependent cytotoxicity (CDC) through Fc region, targeting of an effector molecule, e.g., a cytokine or a toxin, or a prodrug-converting enzyme, retargeting of immune effector cells through a remote binding site, and targeting of drugs loaded nanocarriers. In certain cases, the indirect and direct actions are combined in one system to further enhance its efficacy (Kontermann, 2012). Dual-targeting techniques have a wide range of applications, especially in cancer therapy. Bispecific antibodies can target the same pathways that are exploited in antibody combination treatment. The dual-targeting antibody can thus target and block many disease mediators and signaling pathways at the same time (Chan & Carter, 2010). This comprises targets that act independently on distinct paths, as well as targets that can cross-talk with one another.

## 4. Mechanisms of drugs’ transport across the BBB and design considerations for brain targeted nanocarriers

There are three main mechanisms through which nanocarriers transport therapeutic agents across the BBB. These mechanisms basically use biological characteristics of BBB and can cause: (i) transient opening of BBB caused by “nano-toxicity” or “nano-effects” of the nanocarriers, or stimulus from surface coated surfactants on nanocarriers, leading to diffusion of drugs or drug-loaded carriers into brain parenchyma; (ii) adsorption of drug-loaded carriers on the surface of brain capillary endothelial cells (CECs), with subsequent release of the drug from the vehicle leading to increased drug concentration facilitating gradient and ultimately diffusion of the drugs into the brain; (iii) carrier-mediated transport of the drugs and drug-loaded nanocarriers through endocytosis, trans-cytosis, and/or exocytosis by brain capillaries, resulting in direct penetration to brain cells or parenchyma (Win & Feng, 2005; Zhang et al., 2009; Kreuter, 2013).

It has been reported that chitosan-based nanocarriers can open tight junctions of the BBB (Mao et al., 2010; Vllasaliu et al., 2010), and increased concentrations of some cationic and some anionic nanocarriers can disrupt the BBB. Nanocarriers with neutral surface charge and low concentration anionic nanocarriers have no obvious effect on the integrity of BBB (Lockman et al., 2004). Sodium dodecyl-sulphate (SDS), a biocompatible surfactant, has also been reported to have some disruptive effects on the BBB (Kobiler et al., 1989). The transient opening of BBB induced by surfactants or nanocarriers stimuli is regarded as dangerous because of the toxic nature of nanocarriers as well as due to the diffusion possibility of other substances from blood into the brain at the same time. The adsorption of drug-loaded nanocarriers on the surface of brain CECs is governed by different factors including surface charge, hydrophilicity, and targeting ligands in the nanocarriers system. The lipophilic nature of the carrier and the positively charged surface of the carrier facilitate adsorption as the positively charged carriers can electrostatically interact with the negatively charged endothelial cells' surfaces (Markoutsa et al., 2011; Lien et al., 2012). Certain targeting ligands e.g., apo-lipoproteins decorated on the surface of nanocarriers can target and interact with specific receptors on CECs (i.e., low-density lipoprotein receptors) which ultimately leads to facilitated adsorption (Kim et al., 2007).

The fate of drug-loaded nanocarriers depends on two factors when they are adsorbed on the brain CECs surface including the desorption phenomenon which is followed by reentry into blood circulation. The loaded drug is first released from the nanocarrier on the BBB surface and then traverses into the brain via BBB. Thus, the penetration of drugs to brain parenchyma is mainly determined by drugs' physicochemical characteristics. For better transport of the drug molecules across BBB, it is also important that the surface of the nanocarriers shall be modified to minimize its clearance by macrophages and prolong the blood circulation time and enhance its confrontation with BBB. Increased surface charge and enhanced hydrophilicity are the two good strategies for masking nanocarriers from mono-nuclear phagocyte system (MPS) clearance and increasing their circulation times. Endocytosis by cells is another fate of the adsorbed drug-loaded nanocarriers, often followed by exocytosis which leads to penetration of the drug-loaded carriers to brain cells or parenchyma. Endocytosis could occur by virtue of random uptake by caveolaes on the cells of plasma soluble molecules along with plasma (Simionescu & Simionescu, 1991). Endocytosis of drug-loaded carriers is primarily dependent on their adsorption to the clathrin-coated pit membranes because the brain CECs are have few caveolaes while they have a high density of negatively charged and clathrin-coated pits (Hervé et al., 2008).

Although endocytosis is the normal biological function of cells, it is accomplished by expenditure of energy and thus does not show any preference for drug-loaded nanocarriers leading to low drug transport efficiency across the BBB. Various strategies have been proposed to enhance endocytosis by brain CECs; including nanocarriers’ coating with biocompatible surfactants with proven effectiveness. Surfactants- promoted endocytosis could be explained by three main mechanisms (Juillerat-Jeanneret, 2008) i.e., (i) poloxamers 188- or Tween-80 induces adsorption of apo-lipoproteins E and/or A-I on the nanocarriers surface in plasma which facilitates targeting of low-density lipoprotein receptors on BBB; (ii) surfactants induces fluidity in the cell membrane upon their contact and facilitate disruption in the cell membrane and promote endocytosis; and (iii) surfactants-induced transient opening of the BBB (Li et al., 2017).

Another strategy for provoking endocytosis is to promote adsorption of drug-loaded nanocarriers on brain CECs with the help of transport proteins, specific carriers, or receptors on the cell membrane of the BBB. Relatively higher BBB permeability of acrylic nanoparticles has been observed by some researchers which could be attributed to adsorption of the apo-lipoproteins on nanocarriers’ surface from the plasma which then target low-density lipoprotein receptors on endothelial cells (Göppert & Müller, 2005). A number of nanocarriers have been surface-functionalized with functional peptides such as lactoferrin and transferrin which showed their effectiveness both in *in vivo* and *in vitro* tests in promoting BBB penetration of nanocarriers (Huang et al., 2008). Nevertheless, such specific transport proteins, carriers, or receptors are scarcely expressed on brain CECs membrane, thus attaining minimal effective concentration of therapeutic molecules in brain by such carriers, transporters-, as well as receptor-based endocytosis cannot be guaranteed (Costantino & Boraschi, 2012).

## 5. Nano – based brain cancer targeting

Nanotechnology, in current times, has gained a notable interest and is considered a well-known scaffold for the delivery of therapeutics (Usman et al., 2020). It offers multiple advantages such as ease of preparation with designed functionalities, surface modifications to achieve desired targeting, efficacy improvement through altering the pharmacokinetic profile of therapeutics and its ability to penetrate via leaky vasculature of the tumors (Sonali et al., 2018). As discussed earlier, blood-brain barrier (BBB) is the main hurdle in the way therapeutics enter the brain that ultimately leads to therapy failure (Tu et al., 2021). Luckily, nanocarriers especially gold-based and polymers-based nano-materials (GBNs) have solved that issue which loading therapeutic substances and delivering them to specific brain tissues with enhanced stability, solubility, and reduced side effects (Beik et al., 2019).

In the context of nano-based brain cancer delivery, Ruan et. al., explored doxorubicin-loaded gold nanoparticles using an acid-responsive hydrazine linker. Further, the BBB penetration was mediated through fictionalization with angiopep-2. Both *in-vivo* and *in-vitro* results suggested higher delivery efficiency for gold-based nanoparticles as compared to the free drug (Ruan et al., 2015). For glioma treatment, the prepared gold nanoparticles were grafted on gelatin nanoparticles that mimicked the BBB penetration efficacy during therapy (Ruan et al., 2015). Three chemotherapy drugs decitabine, gemcitabine, and temozolomide were loaded on gold nanoparticles via electrostatic interactions and targeted to U87 human malignant Glial cells following the mechanism of cell-mediated transport. This delivery system integrated the synergistic effect of three drugs and showed a significant therapeutic effect on glioma with a reduction in resistance of glioma cells to temozolomide (Sahli et al., 2020). In addition to drugs, genes delivery was also supported through the aid of gold nanoparticles. In a research study, RNA-based spherical nucleic acid gold nanoparticles were fabricated and evaluated for glioblastomas multiform focusing on oncogene expression regulation. The prepared nano conjugate was administered through intravenous injection in a mice model. Results indicated an enhanced BBB penetration was achieved by gold-based nanoparticles targeting Bcl2-L12 gene knockdown in glioma, consequently resulting in tumor growth inhibition and lengthening the mice survival time (Tommasini-Ghelfi et al., 2019). Bcl2-L12 specific siRNA-loaded gold nanoparticles have entered to preclinical trail phase for recurrent glioblastomas treatment (Kumthekar et al., 2019). Furthermore, probes, photo sensitizers, and antibodies were loaded on gold nano particles and evaluated for brain cancer-targeting (Cheng et al., 2011; Groysbeck et al., 2019). All these findings suggest that gold base nano scaffolds provide a significant and suitable nano platform to deliver therapeutic agents to brain cancer paving the way to overcome the hurdle of the blood-brain barrier.

Quantum dot is another nano platform used for targeting brain tumors. It exhibits certain versatile advantages like low toxicity, easy modifications, fluorescence, small size, biocompatibility, and hydrophilicity (Devi et al., 2019). Carbon quantum dots were fabricated and functionalized with Mal-PEG-NHS linked RGERPPR and targeted to brain cancer cells. The delivered complex captured a clear photo of glioma resulting in efficient penetration of quantum dots to glioma (Gao et al., 2018). Transferrin grafted doxorubicin/paclitaxel loaded mesoprous silica nano particles were fabricated and targeted to glioblastoma. Transferrin acts both as a targeting agent and gatekeeper as well. Results suggested improved doxorubicin cytotoxicity and release as compared to free drug. The tumor growth was efficiently suppressed with reduced systemic side effects (Cui et al., 2013). Similarly, doxorubicin conjugated arginylglycylaspartic acid peptide cRGD were fabricated with various size mesoprous silica nanoparticles (MSNs) and targeted to glioblastoma through blood-brain barrier. Nanoparticles of the conjugate system with 40 nm showed improved cellular uptake. Results also indicated that MSNs functionalization and particle size adjustment are basic parameters that result in efficient targeting of glioblastomas (Mo et al., 2016). The discussion shows that surface modification of MSNs results in efficient targeting, however further studies are required to explore the surface modification for drug release and control release of therapeutic agents.

Polymeric nanoparticles are another scaffold for brain cancer targeting with versatile properties like improved shelf life and release kinetics (Kamaly et al., 2016; Wang et al., 2018). Temozolomide loaded, OX26 type monoclonal antibody functionalized poly lactic-co-glycolic acid nanoparticles were fabricated and targeted to glioblastomas tumor cells. The fabricated nanoparticles were evaluated for targeted cellular internalization and U87 and U215 cell lines were used for *in-vitro* drug cytotoxicity. Results indicated an enhanced temozolomide anticancer activity for fabricated nanoparticles while cellular internalization was mimicked via functionalized monoclonal antibody (Ramalho et al., 2018). In a glioblastoma mice model, miR-296-5p and miR-148a loaded polymeric nanoparticles were targeted to glioblastomas tumor cells and results showed an enhanced and long-term survival in mice with malignant glioblastoma (Lopez-Bertoni et al., 2018). In a recent research study, bevacizumab-loaded poly(D,L-lactic-*co*-glycolic acid) nanoparticles (PLGA NP) were fabricated to circumvent the BBB and off-target side effects at the organ level. The prepared polymeric nanoparticles were evaluated for their pharmacodynamic and pharmacokinetic profiles in CD − 1 mice. Nanoparticles loaded with bevacizumab showed a boosted bioavailability as compared to free drug on one hand and an increased bevacizumab resident time in the brain along with mimicked penetration was observed on another hand (Sousa et al., 2019). In summary polymeric nanoparticles as the carrier could be an efficient strategy to improve survival and quality of life in brain cancer patients. Due to the versatile advantages of polymeric nanoparticles, they can be used in multiple brain cancers to overcome the BBB and streamline the therapeutics to desired targets.

## 6. Dual-targeting in cancers

Dual-targeting strategies focusing on various cancers have been explored in recent studies that have shown that dual-targeting is significantly beneficial over single targeting strategies (Rajendra et al., 2021). Cancer cells especially metastatic ones are continuously changing their microenvironment leading to heterogeneity even in adjacent regions with a varied expression of targetable receptors. Non-targeted nanocarriers are deposited significantly lower in such cancer cells than their dual- or single-ligand targeting nanocarrier counterparts (Covarrubias et al., 2019). For the selection of dual targets in cancer treatment, the two targets should have interdependent characteristics which should collectively enhance the therapeutic efficacy of the delivered drugs. For example, dual-targeted delivery of nanocarriers aimed at targeting α_v_β_3_ integrin and EGFR achieve nearly 2-fold higher deposition in breast cancer metastasis in lungs than single targeted nanocarrier counterparts (Peiris et al., 2018). In a recent study, exosome-based dual-targeted therapy was carried out for the cancer microenvironment. Results showed excellent biocompatibility response for exosomes targeting the immune system and provided a synergistic combination immunotherapy platform (Fan et al., 2021). Similarly, tumor-related macrophages were targeted by dual-targeting zoledronate-loaded biotin- and mannose-modified lipid-coated calcium nanoparticles that targeted macrophage mannose receptors and tumor cells as well. The tumor progression was restrained along with a reduction in angiogenesis in lung cancer cells (Zang et al., 2021). It shows that dual-targeting of tumor cells and their associated macrophages from a nano platform could be a promising approach for lung cancer treatment.

Killing two targets with a single shot rather than killing individual types of cells is the unique beauty of a nano-based dual-targeting system. In a recent study, myeloma cells and cancer fibroblast cells were killed by paclitaxel–loaded poly (ethylene glycol)-poly (lactic acid) nanoparticles with a cyclic peptide targeting platelet-derived growth factor receptor that is over expressed on the surface of both myeloma cells and cancer fibroblast cells. The *in-vivo* antitumor results showed that the efficacy of the dual-targeting system was stronger than free drug and drug – nanoparticles as well (Wang et al., 2021). It could be a promising approach in the clinical translation of myeloma treatment. Similarly, STAT3 and EGFR have been inhibited through a dual-targeting system of Erlotinib + Alantolactone co-loaded poly (lactic-co-glycolic acid) (PLGA) nanoparticles in pancreatic cancer. An ideal anticancer effect by a dual-targeting system was achieved through cell apoptosis as compared to individual co-delivery of two drugs (Bao et al., 2021). Macrophages and intestinal epithelial cells were targeted by Rhein-loaded calcium pectinate – hyaluronic acid – lactoferrin nanoparticles to explore its anti-inflammatory and colonic mucus damage repair effect. Colonic healing was confirmed by alleviation of inflammation through inhibition of the TLR4/MyD88/NF-κB signaling pathway (Luo et al., 2021). Paclitaxel-loaded transferrin and marimastat-loaded thermo sensitive liposomes were fabricated and dual-targeting was carried out to the cancer cell microenvironment. *In-vivo* studies showed the highest tumor growth inhibitory rate as compared to the control group (Zhang et al., 2021).

## 7. Dual-targeting strategies for brain cancer

**Figure 4.**
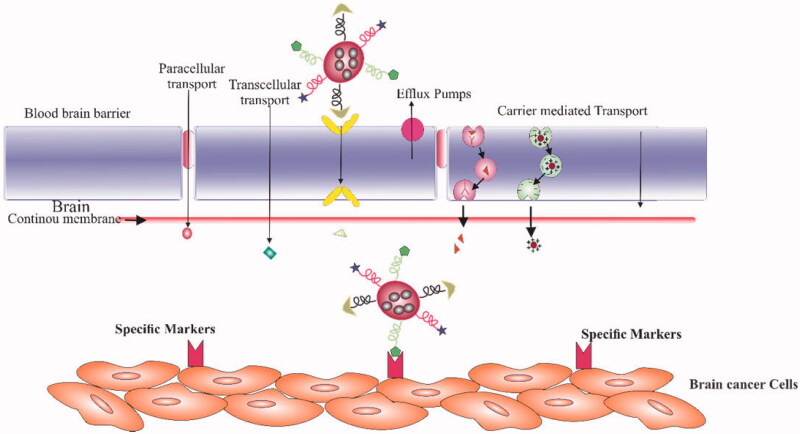
Illustration of a dual-targeting system for brain tumors and transport through BBB. The delivery system function to penetrate through BBB (first barrier) and subsequently target brain tumor cells (second barrier).

As discussed earlier, blood-brain barrier (BBB) is a pressing obstacle in the way of therapeutics targeting brain cancers and tumors. The available treatment drugs suitable to target brain cancers are thus rendered from their therapeutic potential (Karlsson et al., 2021). To overcome such hurdles, a dual-targeting strategy is one of the important obstacle overcoming tools (Deng et al., 2019). The dual-targeting strategy of therapeutic delivery is designed to overcome two barriers encountered in the delivery of drugs to brain tumors. This means that the dual-targeting systems should target two barriers i.e., penetration through or bypassing the BBB and then targeting the brain tumor cells as illustrated in Figure 4. In the context of dual-targeting, to efficiently cross the BBB and target glioma cells need a significant nano carrier system. In this regard, Dual-targeting Temozolomide (TMZ) loaded triblock polymer-coated magnetic nanoparticles were developed and surface modification was carried out through conjugation with folic acid. The purpose of conjugation was to cross the BBB. The duel targeting confirmed by inductively coupled plasma optical emission spectrometry and Prussian blue staining resulted in enhanced penetration across the BBB and the fabricated nano particles were significantly accumulated in brain cells of mice. The effective anticancer potential of dual-targeting temozolomide – folic acid nano particles was confirmed by increased survival time (>100%) with reduced tumor volume (*p* < .001) (Afzalipour et al., 2019). Luo et, al. designed a dual-targeting, novel high-intensity focused ultrasound – responsive angiopep − 2 – modified small poly (lactic-co-glycolic acid) PLGA hybrid nanoparticles loaded with doxorubicin/perfluorooctyl bromide targeting glioblastoma in order to improve the accumulation of used therapeutic drugs in brain cancer mice. The dual-targeting nano carrier-based system showed the strongest antiglioblastoma effect with the longest median survival time of 56 days and reduced vestiges of tumor cells (Luo et al., 2017).

Unsatisfactory accumulation of drugs in brain cancer cells is a challenging hurdle in the respective therapy. In this context, the dual-targeting strategy was adopted for doxorubicin targeting brain tumor cells. A tumor homing peptide was considered in the strategy that exhibit affinity for interleukin receptor (IL-4R) and was conjugated to doxorubicin-loaded PLA nanoparticles. The findings of the study indicated that peptide decorated nanoparticles showed an affinity for both endothelial cells and the tumor environment. The fabricated peptide-functionalized nano particles also showed more accumulation at the tumor site as compared to nanoparticles complex without peptide conjugation (Sun et al., 2017). Transferrin receptor binding peptide and magnetic guidance dual-targeting strategy was developed that was based on T7-mediated active targeting delivery. In addition, PLGA – magnetic nanoparticles system was designed that was based on T-7 modification. Two therapeutic drugs for brain cancer i.e., Curcumin and Paclitaxel were co-encapsulated with the prepared magnetic nanoparticles. Results indicated brain growth inhibition through the synergistic effect of drugs that was more than the individual delivery of drugs. The dual-targeting strategy showed a 10-fold more effect in drugs' cellular uptake and 5-fold more brain targeting as compared to nanoparticles nano targeting. For the anticancer activity magnetic field was provided and the prepared system showed reduced side effects with enhanced therapeutic efficacy (Cui et al., 2016). Similarly, among peptides, L-AE is a targeting peptide that exhibits a high affinity for EGFR (epidermal growth factor receptors) and tumor cells as well. Such peptide-based paclitaxel-loaded micelles were developed and evaluated in the blood-brain tumor barrier/U87 tumor spheroids co-culture model for BBB penetration capability and transcytosis efficiency. Results showed efficient penetration across blood-brain barrier with significant accumulation of the drug in brain cells with effective anticancer activity expanding horizons in brain onco-therapy (Mao et al., 2017). As a whole, it shows that peptide conjugation and fabrication of anticancer therapeutic nanoparticles as a dual-targeting strategy could be used as an effective means of brain cancer therapy.

**Figure 5.**
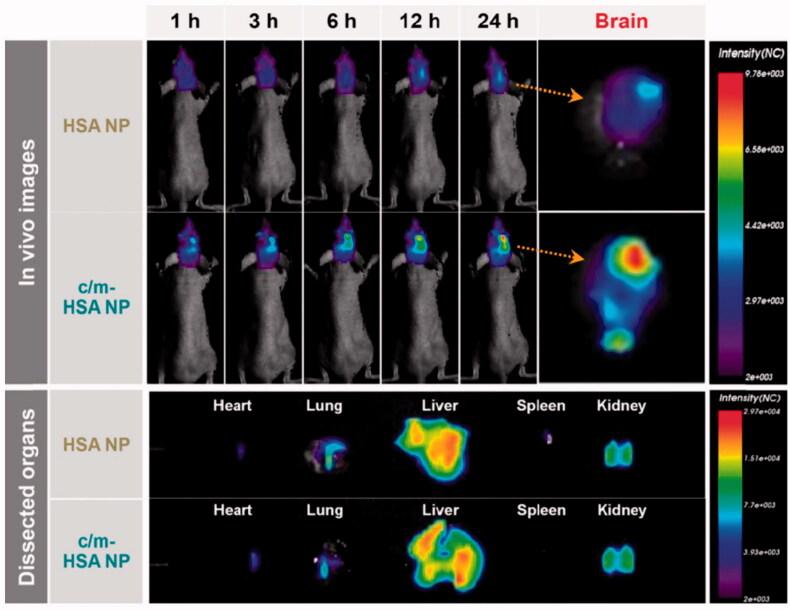
Distribution of HSA nanoparticles and c/m-HSA nanoparticles in glioma-bearing mice (left, upper = after 24 h of intravenous injection; and right upper = ex-vivo image of the brains excised after 24 hours) adapted with permission from (Byeon et al., 2016).

The tumor microenvironment is composed of tumor-associated macrophages. Considering this aspect in tumor cells and BBB, a dual-targeting and biomimetic co-delivery strategy for the treatment of brain tumors was developed. Mannose-modified albumin nanoparticles were fabricated that passed effectively through BBB following the route of nutrient transporters – highly expressed in glioma cells and blood-brain barrier. Co-delivery of this complex system with disulfiram significantly inhibited the proliferation of glioma cells. An improved treatment outcome was observed from the synergistic effect of chemotherapy and immunotherapy based from nano platform (Zhao et al., 2018). Glioblastomas stem cells are considered to show resistance toward therapy strategies such as radiation and chemotherapy resulting in tumor reoccurrence in glioblastomas multiform population. In addition, temozolomide faces the hurdle of BBB during brain tumor therapy. In this regard, temozolomide-loaded dual-targeting immunoliposome were developed and to ensure transcytosis and overcome BBB, anti CD-133 monoclonal antibodies and angiopep-2, respectively, were used along with the nano delivery system. The dual-targeting system showed an *in-vitro* cytotoxicity effect that was 425 fold as compared to the free drug and non-targeted nano delivery system. The median survival time and life span of tumor-bearing mice were increased via a dual-targeting strategy (Kim et al., 2018). Thus showing the effective role of temozolomide-based dual-targeting strategy in the glioblastoma multiform modality. The angiopep-2 modification in the brain was also found to indulge in gene expression elevation when adopted for dual-targeting using dendrimers and liposomes as nano carrier delivery systems (Gao, 2017). Cationic and mannose modified doxorubicin-loaded albumin nanoparticles were synthesized resulting in dual-targeting by penetrating BBB and targeting brain tumors following the mechanism of GLUT and absorptive mediated endocytosis (Byeon et al., 2016). In U87 brain tumor cells, the prepared dual-targeting delivery system posed prominent performance. The *in-vitro* and *in-vivo* results are shown in Figure 5.

To improve the chemotherapy efficacy, using a dual-targeting strategy the brain tumor immune environment was modified using RNAi-based modulation through the development of dual-targeting nano theranostics system for glioblastomas treatment. Results indicated high serum stability, strong condensation, and significant loading efficiency for siRNA. In addition, through receptor-mediated transcytosis, the prepared nano particles cross BBB and efficiently targeted brain tumor cells (Qiao et al., 2018). To explore the dual-targeting delivery from nano platform for glioblastomas treatment, a dual-targeting delivery system based on VLP (protein-virus-like particle) for overcoming the hurdle of BBB and efficient targeting of brain tumor. Therapeutic agents – siRNA and paclitaxel were co-packed inside the delivery system. Results showed an efficient delivery of payloads to the desired target site i.e., invasive tumor site. The apoptosis and necrosis were enhanced by the synergistic effect of the gene and chemotherapy. Minimal cytotoxicity was observed during the course of tumor invasion inhibition (Yang et al., 2020). In the context of RNAi dual-targeting to brain tumors, cationic liposomes were developed using RNA aptamers and LDL receptor-related protein dual-targeting ligands. The prepared conjugated system was loaded with paclitaxel and survivin siRNA and targeted to brain tumor cells. *In-vitro* results suggested apoptosis of brain stem cells with efficient penetration across the blood-brain barrier confirmed via significant accumulation of therapeutic agents in the tumor cells (Fu et al., 2019). Overall, the results suggest the role of siRNA-based dual-targeting strategy from nano carrier scaffolds paves the hurdles in brain tumor therapy with special emphasis on chemotherapy efficacy and blood-brain barrier.

In addition to BBB, blood-brain tumor barrier (BBTB) is another hurdle that is a prominent obstacle in the delivery of chemotherapeutics to brain tumor cells (Chen et al., 2019). In this regard, doxorubicin-loaded micelle with a dual-targeting pattern was designed and targeted to a brain tumor in a mice model. Results were in favor of the dual-targeting system as compared to pure delivery of the conventional drug–carrier system. *In-vivo* pharmacokinetic studies showed a 5-fold increase in bioavailability for a dual-targeting system as compared to a controlled applied solution. The survival time for the drug-loaded dual-targeting system was 32 days as compared to 19 days of the pure drug suggesting an improved efficacy for doxorubicin dual-targeted delivery by overcoming the BBTB (Niu et al., 2020). Cui et, al. worked on a dual-targeting strategy targeting glioma by focusing blood-brain tumor barrier remains a significant challenge in the treatment of brain tumor therapy. Euphorbia factor L1 (extracted from euphorbiae semen) – loaded erythrocyte membrane coated PLGA nanoparticles were developed and evaluated for *in-vitro* and *in-vivo* characterization. Results of the *in-vitro* studies showed an enhanced cytotoxic effect by the fabricated dual-targeting system through efficient penetration across BBB and BBTB. Whereas, the *in-vivo* results of the specialized nano-based dual-targeting system showed an extended survival time with significant penetration and accumulation of the drug in the brain glioma cells (Cui et al., 2020). Imaging of brain tumor cells has a direct association with tumor therapy. Quantum dots and magnetic iron oxide-loaded nanoparticles were loaded niosomes decorated with transferrin. *In-vitro* imaging studies for the developed dual-targeting system showed improved fluorescence intensity and negative-contrast enhancement effect on glioma cells representing its promising dual-targeting imaging niosome platform (Ag Seleci et al., 2021). Some other studies carried out on dual-targeting strategies for a brain tumor are shown in Table 1.

**Table 1.** Dual targeting strategy for a brain tumor.

Therapeutic agent	Carrier system	Targeting pattern	References
Paclitaxel	Polyester based nanoparticles	Tumor cells and BBB	Di Mauro et al., 2018
Borneol and doxorubicin	Dendrimers	Tumor cells and BBB	Xu et al., 2016
pORF-hTRAIL, pGL2	Nanoparticles	Dual targeting to tumor cells and BBB	Huang et al., 2011
Docetaxel	Dendritic nano conjugates	Targeting BBB for efficient delivery to tumor cells	Gajbhiye & Jain, 2011
HSV-TK plasmid	Polymeric nano particles	Dual targeting to BBB and Tumor cells	Gao et al., 2016
siRNA	Polyester dendrimers	Targeting BBB	Stenström et al., 2018
Irinotecan and gadolinium-diethylenetriamine pentaacetic acid	Crosslinked hyaluronic acid nanoparticles	Dual targeting of BBB and brain tumor cells along with boosted glioma imaging	Costagliola di Polidoro et al., 2021
Doxorubicin	Nano gel	Dual targeting of BBB and glioma cells	Liu et al., 2021
Dbait (a small double- stranded DNA)	Nano micelle	Dual targeting of BBB and glioma cells	Jiao et al., 2019

## 8. Challenges in design of targeted drug delivery

The advantage of dual-targeting is the expected manifold increase in the therapeutic efficacy of treatment because multiple interdependent processes are affected leading to the augmented overall response. Although there has been much advancement seen in the design of targeted delivery systems, still there are various challenges that restrict the successful clinical translation of targeted therapies. Receptors' specific challenges include troubles in receptors identification, varied expression properties, accessibility of receptors in terms of availability and access, and receptor shedding (Tewabe et al., 2021). Challenges associated with ligands include proper selection of ligands, designing of strategies for conjugation of ligands with carrier/drug, and drug release characteristics (linker selection) from ligands. Selection of carrier, pharmacokinetic and physicochemical characterization of nanocarriers are some of the challenges associated with carrier selection (Vhora et al., 2014). In addition, there are a number of rough facts that are misconceived and overlooked regarding dual-targeted drug delivery. The first and foremost is that targeting is not perfect and precise, rather it implies simple distribution. The second is the incomplete correlation of the theory of receptors overexpression and targeted delivery. In addition, improved delivery has been observed with the EPR effect, but not such precise with targeted delivery. Moreover, the drugs may be prematurely released from the targeted delivery system before reaching the target tissue and tumor thus does not guarantee improved delivery (Kwon et al., 2012).

Additional steps in the synthesis and purification of targeted drugs’ formulations are needed. Accordingly, more regulatory and quality control steps are needed, the cost is increased, and the developmental time lines are prolonged. Biocompatibility, safety, sensitivity, and scalability are all always the design associated challenges for nanocarriers (Rosenblum et al., 2018; Yoo et al., 2019). Additional complexity arises from the immense heterogenic nature of tumors and the existence of tumor- and metastasis-associated fibroblasts and macrophages (Rosenblum et al., 2018). The lack of completely specific targets and clinically translatable models still makes the practical outcome of drug-targeting strategies debatable. A noteworthy barrier to the translation of nano-medicines into human clinical medicine is made by the unverified EPR effect in human clinical oncology, the lower NPs accumulation within tumors with active targeting mechanisms contrary to the expectations, and many other factors that should be controlled, considered, and modified during preparation targeted nanomedicines (Sindhwani et al., 2020).

Targeting using nanocarriers is dependent on particle size, hydrophobicity, surface modification, and surface charge. There is still much to learn about nanocarrier’s toxicity, ligands toxicity and fare, and other challenges with selective binding and targeted administration. Considering these issues today may lead to more fruitful therapeutic and research paradigms in the future (Rosenblum et al., 2018). The future outlook for addressing these issues and maximizing targeted delivery is brightening. Some are briefly stated below. The clinical extrapolation of targeted systems is still lacking. This requires improved methodologies, reproducible carrier preparation procedures, and rigorous preclinical studies (Vhora et al., 2014).

Receptor-targeted delivery has great promise for discovering new therapeutic targets, developing enhanced biological products, and developing NDDSs for cell-specific delivery (Vhora et al., 2014). Nanomedicine's future will merge diagnosis and tailored therapy into a single treatment system. This unique theranostic technique may result in tailored chemotherapy with improved patient outcomes. The creation of targeting strategies should be continually evaluated in light of new knowledge about post-administration processes. 16 Greater preclinical animal models, a better understanding of tumor biology, and identification of genuine biomarkers will speed up clinical translation (Rosenblum et al., 2018).

## 9. Conclusion and future perspectives

Advances in molecular neuroscience and nanocarrier-based drug delivery platforms have changed the deployment of nanotechnology-based techniques for the improved treatment of metastatic brain cancers. Nanocarrier-based drug delivery platforms are projected to reach new heights in the next years, bringing about significant improvements in oncology research. As the five-year survival rate of brain cancer patients remains low, successful clinical translation of nanocarriers is critical. Nano drug delivery systems for the brain must be well-characterized, in terms of safety, biocompatibility, biodegradability, and, of course, must be intelligent in order to function well in in vivo conditions. However, it must be target-specific, i.e., it must be concentrated in brain tissue and successfully traverse the BBB while avoiding off-target drug release in order to have a maximum therapeutic impact with minimal side effects.

To target brain tumors, several dual-targeting delivery strategies have been developed. In general, such strategies are intended to traverse the BBB while targeting brain tumor cells or other stroma cells in brain tumors. Dual-targeting delivery systems have been shown to deliver more drug concentration to brain tumors, resulting in increased anti-tumor efficacy in vivo. More research should be done in the future to determine the effect of various parameters such as ligand density and ratio, linker length, particle shape, and size on the delivery effectiveness of dual drug targeting delivery systems. Despite the fact that dual-targeted delivery systems are still in their infancy, clinical translation of such systems appears to be a common trend. However, a number of challenges and concerns must be resolved before translational research may begin. Because safety is the utmost concern for clinical application, nanocarriers of biodegradable nature such as liposomes, albumin-based, and polylactic acid (PLA)-based NPs are advised (Weissig et al., 2014). Another issue is the preparation approach, because laboratory procedures, particularly for ligand modification, are unsuitable for large-scale production. It is suggested that ligands be conjugated with materials prior to the formulation of a nano-delivery system, and numerous prospective nanomedicines employing this technology are currently being evaluated in clinical trials (Meel et al., 2016).
